# Geogenic and anthropogenic interactions at a former Sb mine: environmental impacts of As and Sb

**DOI:** 10.1007/s10653-020-00652-w

**Published:** 2020-07-07

**Authors:** Lenka Mbadugha, Duncan Cowper, Sapar Dossanov, Graeme I. Paton

**Affiliations:** School of Biological Sciences, University of Aberdeen, Cruickshank Building, St. Machar Drive, Aberdeen, AB24 3UU Scotland UK

**Keywords:** Arsenic, Antimony, Abandoned mine, Contamination, Pollution, Risk assessment

## Abstract

Mining activities are acknowledged to introduce contaminants into localised environments and cause wider spread diffuse pollution. The concentration, distribution and fate of arsenic (As) and antimony (Sb) were studied at the former metalliferous Louisa Mine at Glendinning, Scotland. Soils and surface water were sampled and subsequently analysed to map the distribution of contamination and identify pollution sources. The maximum concentrations of As and Sb of 15,490 and 1504.2 mg kg^−1^, respectively, were determined in soils associated with the ore processing area and spoil heaps. The fractions of dissolved As and Sb in soils were < 1 and < 5% of total soil content, respectively, confirming findings of previous studies that As and Sb are relatively immobile. Yet, the concentrations of As and Sb released by soils exceeded regulatory limits. Concentrations of As and Sb in surface water in the immediate vicinity of the mine were impacted by a gully discharge, but rapidly diluted. While the concentrations affected by the run-off waters did not exceed EU environmental standards for freshwater, the concentrations of both, As and Sb, sharply increased above the said environmental standards approximately 100 m downstream of the mine site. The unaltered As-to-Sb ratio in water samples suggests a geogenic source. While there is a justifiable concern about the soil pollution caused by the historic mining in the area, the Glenshanna Burn is affected more by indigenous geochemical processes than the derelict mine.

## Introduction

Arsenic (As) and antimony (Sb) are potentially toxic elements (PTEs) ubiquitously present in the environment (Wilson et al. 2010). They often occur together and enter the environment through natural geogenic processes, such as volcanic emissions or mineral weathering (Tan et al. 2018). Although the natural sources account for the majority of elevated As and Sb concentrations in the environment, anthropogenic activities, such as mining operations, metal processing, agriculture and the combustion of fossil fuels, can be locally important (Fei et al. 2017; Hiller et al. 2012). Antimony mining, in particular, has been recognised as a major anthropogenic source of As and Sb contamination (Borčinová Radková et al., 2020; Müller et al. 2007; Warnken et al. 2017; Zhang et al. 2018), not only due to current activities, but also due to the legacy of contaminated sites.

Derelict antimony mine sites often represent a serious environmental threat, because their unmanaged wastes continue to release contaminants (Fu et al. 2016; Ondrejková et al. 2013). Soils of the abandoned Sb mining areas tend to be severely contaminated with the concentrations of As and Sb significantly elevated above the natural background levels (Hammel et al. 2000; Wilson et al. 2010). This is of a particular concern, when the soils are cultivated or used for grazing (Álvarez-Ayuso et al. 2012), as a number of pasture plant species and crops have been reported to accumulate unsafe concentrations of As and Sb in their tissues (Abad-Valle et al. 2018).

Due to precipitation, As and Sb can be also mobilised from soils to surface waters and groundwater (Nannoni et al. 2011; Hiller et al. 2012). This pathway represents another serious environmental problem, because it may cause a deterioration of water quality and introduce these contaminants to the food chain with risks to both human health and ecosystems (Abad-Valle et al. 2018; Fei et al. 2017; Protano and Nannoni 2018). Chronic exposure to water contaminated with As has been linked to deleterious effects on human health (Smedley and Kinningburgh 2002). Similarly to As, Sb is clastogenic in the trivalent state and is potentially carcinogenic (Telford et al. 2009).

The potential risk As and Sb present to the environment and human health, has resulted in their classification as “pollutants of priority interest” by both the European Union (EU) and the United States Environment Protection Agency (USEPA) (Gál et al. 2007; Hiller et al. 2012). Critical limits for As and Sb have been recommended for the risk assessment of soil contamination within the EU with aim to protect either human or environmental health (50–100 and 10–50 mg kg^−1^, respectively) (Tóth et al. 2016). The lower concentrations of these critical ranges have been suggested for the assessment of agricultural sites (Álvarez-Ayuso et al. 2012). In addition, the World Health Organisation (WHO) has set guideline environmental standards (ES) for the maximum As and Sb concentrations in freshwater (10 and 20 µg l^−1^ for As and Sb, respectively). However, there are still many uncertainties associated with Sb behaviour in the environment (Telford et al. 2009), as it is a “contaminant of emerging concern”. This uncertainty is reflected in a more conservative EU environmental limit for Sb in freshwater, which has been set to 5 µg l^−1^ (Ondrejková et al. 2013). Given that relatively low concentrations of As and Sb can have detrimental effects on human and environmental health, it is of utmost importance to characterise their spatial distribution and exposure profiles in the areas highlighted as potentially contaminated (Protano and Nannoni 2018).

The aim of this study was to assess risks associated with As and Sb concentrations in soils and surface waters in the vicinity of derelict antimony Louisa mine, Glendinning, Scotland. The area has already received notable interest, with studies investigating mobility and bioavailability of Sb, as well as As and Pb (for example Flynn et al. 2003; Gál et al. 2007; Macgregor et al. 2015). The main objectives of this study were: (1) to evaluate the impact of mining activities on As and Sb soil concentrations; (2) to determine the spatial distribution of As and Sb contamination of soils; (3) to evaluate the impact of soil contamination on the freshwater quality, with the Glenshanna Burn being the receptor, and (4) to translate the observed risks to scenarios beyond the mining site.

## Materials and methods

### Study area

The remnants of the antimony Louisa (Glendinning) mine, one of very few mines able to produce Sb in workable quantities in the UK, are situated near Jamestown in Dumfries and Galloway, South-West Scotland (Gallagher et al. 1983; Macgregor et al. 2015). The underlying geology consists of intensely folded and faulted mudstones, greywackes and intraformational breccias formed during the Caledonian orogeny (Gallagher et al. 1983). The geochemical mineralisation is strongly associated with the breccias quartz veins formed by hydrothermal activity occurring in three phases. The first phase fluids formed pyrite (FeS_2_) and arsenopyrite (FeAsS). The second phase produced stibnite (Sb_2_S_3_), galena (PbS) and sphalerite ((Zn,Fe)S). The final minor phase formed galena, sphalerite, chalcopyrite (CuFeS_2_) and barite (BaSO_4_) (Duller et al. 1997).

**Fig. 1:**
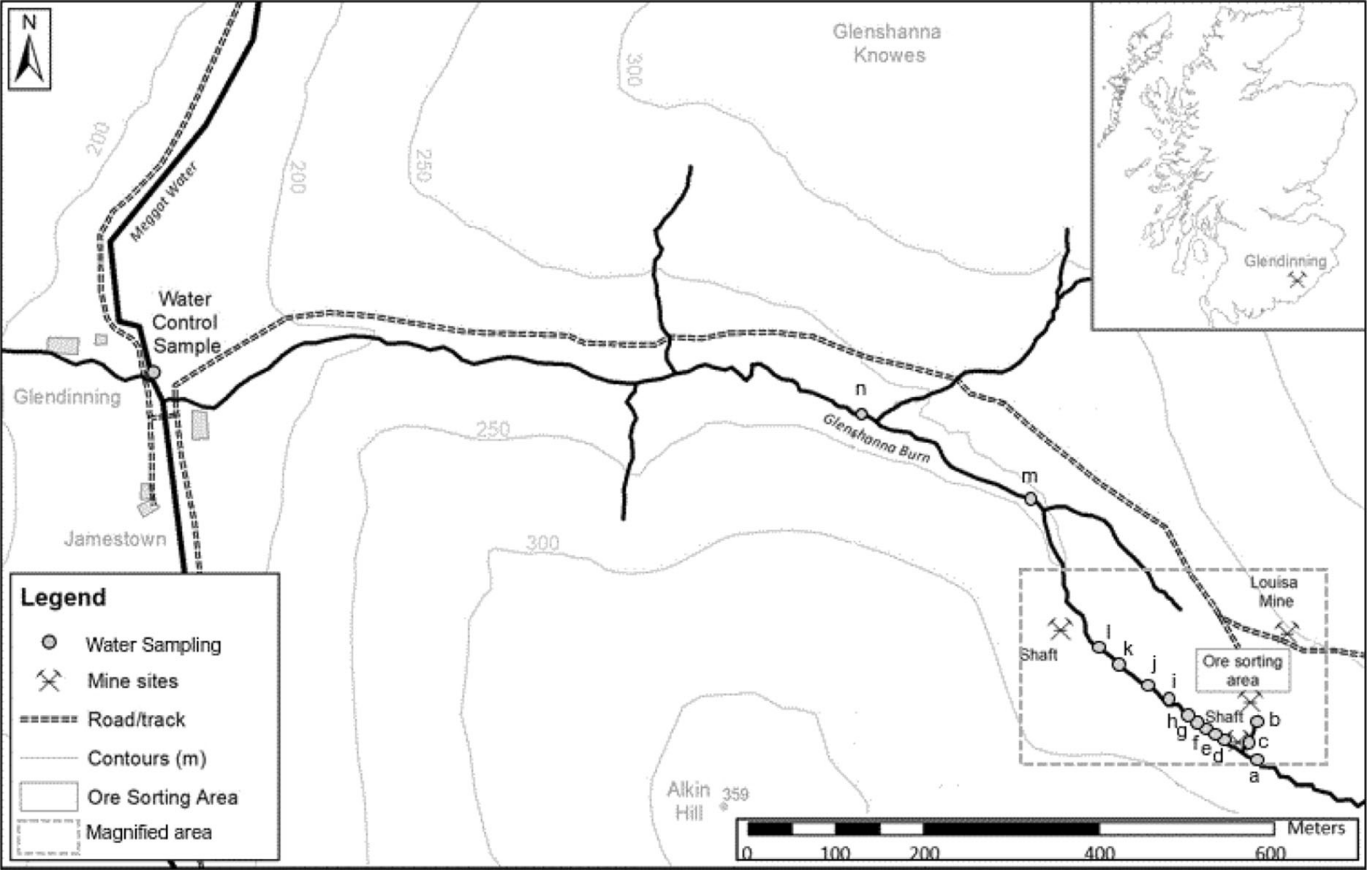
Map of the Glendinning area with the Louisa Mine site, adjacent Glenshanna Burn and water sampling points

The first recorded discovery of Sb in the form of stibnite at Glendinning dates to 1760 with a subsequent exploitation during three brief periods between 1793 and 1922 (Macgregor et al. 2015). In total, approximately 200 tonnes of Sb has been recovered from the site (Gallagher et al. 1983). While the presence of As and other elements, such as lead (Pb) and copper (Cu), has also been recognised (Duller et al. 1997; Flynn et al. 2003), the deposits were never considered economically viable for extraction. The site, which was abandoned without remediation, currently consists of the main mine adit and two spoil heaps adjacent to the former ore processing area (Fig. 1) and is used for grazing (Gál et al. 2007; Macgregor et al. 2015).

### Sampling strategy

#### Soil sample collection and characterisation

A total of forty sampling locations, complementing the previous campaigns by Flynn et al. (2003) and Macgregor et al. (2015), were distributed over the study area (Fig. 1). The sampling locations no. 1–37 targeted spoil heaps, ore processing area, as well as surrounding soils. In addition, three samples were taken at a location of a different shaft (locations no. 38–40) (Fig. 1). The positions of the sampling locations were recorded by Ashtech ASH111660 Survey antenna (NavtechGPS, Springfield, US). Soils were sampled to a 10 cm depth (Gál et al. 2007) and stored in labelled polyethylene Ziplock bags.

Once in laboratory, the samples were air-dried, sieved through a 2-mm sieve and ball-milled for 3 min using a mixer mill MM200 (Retsch, Haan, Germany). Soil pH was measured in both deionised water (diH_2_O) and 0.01 M CaCl_2_ (exchangeable acidity) at a soil/solution ratio of 1:2.5 (w/v). The percentage of the soil organic matter (OM), i.e. the organic component of soil, was determined in oven-dried soils (at 105 °C overnight) by a loss-on-ignition at 375 °C for 16 h. Total Sb and As content of soils was determined by the HNO_3_ and H_2_O_2_ total digest method. Briefly, 0.2 g subsamples of milled soils were weighed into 100 ml digestion tubes and placed in a digestion block. Subsequently, a 2.5 ml aliquot of 70% HNO_3_ (Fisher Scientific, Loughborough, UK) was added to each tube. Following an overnight incubation (16 h) and addition of 2.5 ml of hydrogen peroxide (H_2_O_2_, Fisher Scientific, Loughborough, UK) to each tube, the digestion block was placed in an oven and heated for 1 h at 100 °C, followed by 1 h at 120 °C and 2 h at 140 °C. Once cooled, the samples were flushed from the digest tubes into 50 ml centrifuge tubes using diH_2_O, diluted to make up 50 ml and stored at 4 °C prior to analysis. Quality control was confirmed with the use of certified soil reference material NCS DC 73319 (China National Analysis Center for Iron and Steel, Beijing, China); analytical recoveries > 97% and < 104% were yielded. The mobility of As and Sb in soils was assessed by short-term batch method ASTM D4319-93 at a soil/solution ratio of 1:4 (w/v).

#### Water sample collection and characterisation

Water sampling locations (a and d-n, *n* = 12) complemented the soil sampling campaign and covered a 0.7-km linear transect of the adjacent non-seasonal stream, the Glenshanna Burn. The samples were collected upstream, downstream and adjacent to the mine site (Fig. 1). In addition, to enable a comparison with the most recent sampling campaign by Macgregor et al. (2015), two sampling points were also selected on a small gully flowing into the Glenshanna Burn (locations b and c). A control water sample was collected from the Meggat Water upstream of the Glenshanna Burn inflow (Fig. 1). At each sampling point, recorded by the GPS as detailed above, three independent replicate samples were taken in 50-ml polypropylene centrifuge tubes. Upon arrival to laboratory, water samples were filtered (0.45 μm EDM Millipore Millex Nonsterile 33 mm filters, Millipore, Watford, UK) and subjected to a pH measurement. All water samples were subsequently subsampled into clean centrifuge tubes, acidified with a drop of HNO_3_ and, together with the original samples, stored at 4 °C prior to analysis.

### Instrumental analysis

Total concentrations of Sb and As in the soil digests were determined by inductively coupled plasma-mass spectrometry (ICP-MS) (Agilent 7900, Agilent Technologies, Stockport, UK) using internal standards prepared from certified standard solutions (1000 mg (As) l^−1^ and 1000 (Sb) mg l^−1^, Fisher Scientific, Loughborough, UK). Soil digests were initially diluted 1:100 with 1% HNO_3_ in Milli-Q Integral Ultrapure Water (18.2 MΩ) (reverse osmosis water: ROH_2_O). Where necessary, digests were further diluted to 1:1000 (v/v). The pH values of soils (diH_2_O and 0.01 M CaCl_2_, 1:2.5 w/v ratio) and water samples were measured by HI 1110B pH electrode (Hanna pH 20, Hanna Instruments Ltd., Leighton Buzzard, UK). Dissolved organic carbon (DOC) was analysed by a LabTOC aqueous carbon analyser (Pollution and Process Monitoring, Kent, UK). The consistency of other selected freshwater analytes (chlorine (Cl^−^), fluorine (F^−^), bromine (Br^−^), nitrate (NO_3_^−^), phosphate (PO_4_^3−^) and sulphate (SO_4_^2−^) was analysed using Dionex ICS-90 ION Chromatography System (Thermo Scientific, Loughborough, UK). The total concentrations of As and Sb in the water samples were determined by hydride generation atomic absorption spectrometry (HG-AAS) using AAnalyst 300 (Perkin Elmer, USA) according to Ulusoy et al. (2011). Prior to analysis, As in 5 ml aliquots of acidified water samples was reduced to arsenite (AsO_2_^−^) by 5 ml of a reagent solution containing 20% HCl, 20% KI and 5% ascorbic acid. Gaseous hydrides were generated in a continuous flow system using 10% KI and 0.2% sodium borohydride (NaBH_4_). The carrier gas was argon. Calibration standards were prepared from the same standard solutions used during the ICP-MS analysis. Further quality control was applied by preparing independent standards from a different batch of standard solutions (1000 mg (As) l^−1^, Fisher Scientific, and 1000 mg (Sb) l^−1^ Sb (Sigma-Aldrich, Dorset, UK). A sequential dilution was undertaken on both sets of standards, initially using ROH_2_O and 20% KI solution for the final dilution. A subset of the replicate water samples representing a range of As and Sb concentrations was analysed on the ICP-MS to test the results for both methods of analysis and to provide internal quality assurance. Both methods were in good agreement (*r*^2^ = 0.98). Accuracy of both methods based on independent standards of 10 and 5 µg l^−1^, for As and Sb, respectively, yielded variations within the range of ± 10%. The precision of As and Sb analyses did not exceed ± 5%.

### Environmental risk and pollution assessment

The generic environmental risks associated with soil contamination were expressed against the lower As and Sb guideline values detailed by Tóth et al. (2016): 50 and 10 mg kg^−1^, respectively, recommended for the agricultural sites including pastures. The assessment of risks posed by As and Sb in freshwater samples was based on the EU environmental standards (10 and 5 µg l^−1^ for As and Sb, respectively) (Ondrejková et al. 2013). Total As and Sb concentrations in relevant samples above these values were considered hazardous to human and environmental health.

The extent of pollution of As and Sb was also assessed using contamination factors (CF) and integrated pollution indexes (IPI) according to Guo et al. (2017). The contamination factors, which highlight the intensity of contamination, were calculated by dividing total As and Sb concentrations by their background concentrations in soils. The average soil background As and Sb concentrations were adopted from Gallagher et al. (1983) and approximated the maximum As and Sb background concentrations associated with the mining site (Protano and Nannoni 2018). Subsequently, the overall contamination of the site was classified by four categories: low (CF < 1), moderate (1 ≤ CF < 3), considerable (3 ≤ CF < 6) and high (CF ≥ 6). The integrated pollution indexes signifying the overall pollution status of the site were derived as:

$${\text{IPI}} = \left( {{\text{CF}}\left( {\text{As}} \right) \, \times {\text{CF}}\left( {\text{Sb}} \right)} \right)^{1/2}$$

Similarly to CF, using the IPI the site pollution was classed as either moderate (1 < IPI ≤ 2), heavy (2 < IPI ≤ 3) or extreme (3 < IPI) (Guo et al. 2017). The IPI values ≤ 1 indicated no pollution.

### Spatial and statistical data analysis

**Fig. 2:**
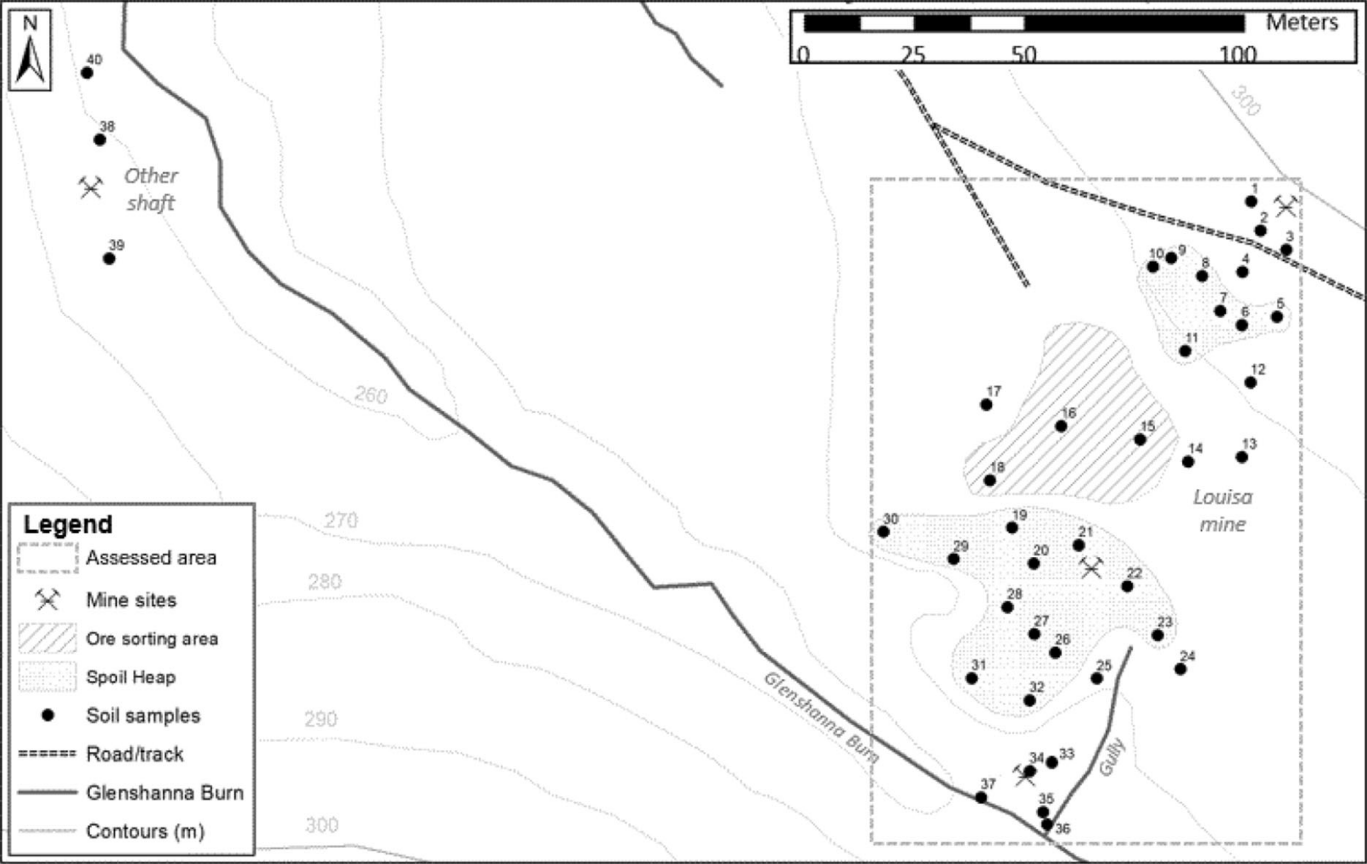
Map of the mine site highlighting the location of spoils, processing area and soil sampling points (filled circle) with sample ID. The ‘assessed area’ outlines the part of site analysed for the spatial distribution of As and Sb contamination and pollution (provided in Fig. 5)

The mobility of As and Sb in soils was evaluated by calculating the soil-solution partitioning coefficients Kd (l kg^−1^) according to Sauvé et al. (2000), whereby As and Sb soil concentrations (mg kg^−1^) were divided by As and Sb concentrations (mg l^−1^) in soil water extracts. The spatial distribution of pollution across the assessed area (highlighted in Fig. 2) was visualised by a kriging method provided by Surfer v 15.0 software (Golden Software, Golden, Colorado, USA). The assessment of anthropogenic redistribution of As and Sb in the studied environmental matrixes (soils and freshwater) was further facilitated by the As/Sb ratios. The ratios were obtained by dividing total As concentrations by total Sb concentrations of the relevant matrix. The ratios that significantly differed from As/Sb ratios in control samples (freshwater) or surrounding soils (soils) were adopted as evidence of anthropogenic redistribution of As and Sb in that matrix. Statistical analysis of data was carried out using Minitab v 19.0 (Minitab, Coventry, UK). Pearson correlation coefficient was used to assess relationships between various soil and water parameters. Kruskal–Wallis test was subsequently used to test the significance of spatial variation of As and Sb pollution. All significant levels were quoted at the 95% confidence level (*p* ≤ 0.05).

## Results

### Characteristics of soil samples

Total soil As concentrations associated with the main site (locations no. 1–37) ranged from 108.33 to 15,490.74 mg kg^−1^. The minimum and maximum concentrations of Sb were 1.15 and 1504.17 mg kg^−1^, respectively (Table 1). The As and Sb concentrations significantly varied on a spatial basis. The As concentrations in the soils surrounding the site averaged 361.67 ± 69.39 mg kg^−1^, but the As content of spoils and ore processing area was significantly higher (*p* < 0.05) (Table 2). While the As concentrations of ore processing area were, on average, 8 times higher, the highest As concentrations were associated with spoils (5768.14 ± 811.92 mg kg^−1^, 16 times higher than the surrounding soils). The maximum As concentration observed at the smaller shaft (locations no. 38–40, Fig. 2) was 747.09 mg kg^−1^. In contrast, the highest concentrations of Sb were associated with the site’s ore processing area (Table 2). The Sb content of spoils averaged 35.69 ± 6.98 mg kg^−1^. The Sb concentrations in surrounding soils were significantly lower (9.91 ± 6.49 mg kg^−1^, *p* < 0.05). The soils sampled at the smaller shaft contained up to 158.51 mg (Sb) kg^−1^. A significant relationship between As and Sb was observed only in the soils surrounding the site (*r*^2^ = 0.69, *p* < 0.05).

**Table 1:** Basic physico-chemical properties of soils

Soil ID	Location	As (mg kg^−1^)	Sb (mg kg^−1^)	pH_aq_	OM (%)	Soil ID	Location	As (mg kg^−1^)	Sb (mg kg^−1^)	pH_aq_	OM (%)
1	Surrounding	376.5	3.50	4.12	2.66	21	Lower spoil	12,960	21.64	5.14	1.89
2	Surrounding	692.4	6.14	3.57	2.63	22	Lower spoil	5134	27.37	5.30	1.78
3	Surrounding	1102	7.91	4.28	1.97	23	Lower spoil	5523	56.12	6.04	1.65
4	Surrounding	321.1	3.59	3.77	4.15	24	Surrounding	269.1	3.18	4.10	3.82
5	Upper spoil	5232	54.28	4.99	2.62	25	Surrounding	293.9	2.20	4.62	1.97
6	Upper spoil	3474	30.74	4.85	3.27	26	Lower spoil	5329	10.06	6.77	0.55
7	Upper spoil	7209	25.56	5.42	1.96	27	Lower spoil	5807	13.96	6.27	0.62
8	Upper spoil	2171	7.46	4.77	1.82	28	Lower spoil	8062	33.45	6.64	0.34
9	Upper spoil	5890	45.14	6.13	1.39	29	Lower spoil	1433	65.31	4.64	4.79
10	Upper spoil	5393	33.09	4.88	2.36	30	Lower spoil	3570	4.36	6.90	0.51
11	Upper spoil	7649	138.1	6.13	5.01	31	Lower spoil	4441	25.17	4.78	3.25
12	Surrounding	355.2	6.05	4.87	2.41	32	Lower spoil	3160	4.41	5.94	3.41
13	Surrounding	275.2	2.41	4.57	4.73	33	Lower spoil	489.3	1.15	4.59	1.43
14	Surrounding	193.4	5.17	4.55	3.54	34	Lower spoil	108.3	1.93	5.29	1.59
15	Processing	3010	236.1	3.99	3.95	35	Surrounding	244.2	4.47	5.09	2.43
16	Processing	6162	288.2	5.27	3.00	36	Surrounding	200.3	1.51	6.79	0.89
17	Processing	426.4	97.44	5.31	4.70	37	Surrounding	142.7	1.98	6.39	0.84
18	Processing	1903	1504	6.34	10.83	38	Smaller shaft	747.1	158.5	5.64	3.44
19	Lower spoil	15,491	30.89	7.08	0.55	39	Smaller shaft	112.2	9.79	4.05	3.40
20	Lower spoil	1664	31.92	3.83	3.21	40	Smaller shaft	20.26	0.45	4.46	2.24

Note: The given values are based on individual samples

**Table 2:** Descriptive statistics of basic soil properties in different zones of Louisa Mine site

Zone	Property	Units	Minimum	Maximum	Mean	Median	Standard error	Count
Upper spoil	As	mg kg^−1^	2171.92	7649.02	5288.78	5393.27	734.25	7
	Sb	mg kg^−1^	7.46	138.14	47.77	33.09	16.07	7
	pH (aq.)		4.77	6.13	5.31	4.99	0.23	7
	OM	%	1.39	5.01	2.63	2.36	0.46	7
Lower spoil	As	mg kg^−1^	1433.31	15,490.74	6047.76	5231.55	1231.73	12
	Sb	mg kg^−1^	4.36	65.31	27.05	26.27	5.44	12
	pH (aq.)		3.83	7.08	5.78	5.99	0.30	12
	OM	%	0.34	4.79	1.87	1.71	0.43	12
Processing area	As	mg kg^−1^	426.42	6161.76	2875.64	2457.19	1216.58	4
	Sb	mg kg^−1^	97.44	1504.17	531.47	262.13	326.72	4
	pH (aq.)		3.99	6.34	5.23	5.29	0.48	4
	OM	%	3.00	10.83	5.62	4.32	1.77	4
Surrounding soils	As	mg kg^−1^	108.33	1101.73	361.67	284.54	69.39	14
	Sb	mg kg^−1^	1.15	7.91	3.66	3.34	0.54	14
	pH (aq.)		3.57	6.79	4.76	4.58	0.24	14
	OM	%	0.84	4.73	2.51	2.42	0.32	14

**Fig. 3:**
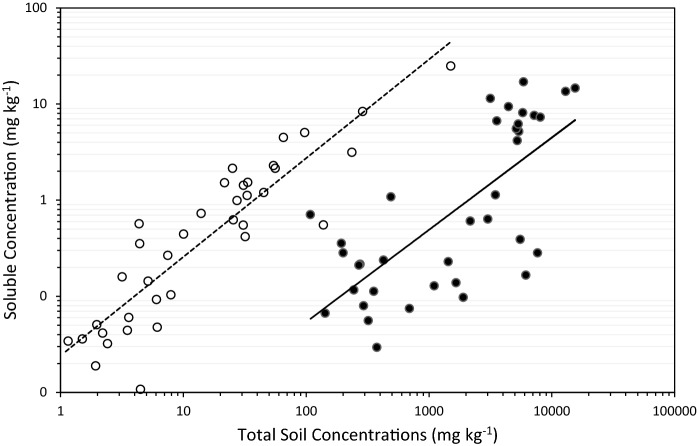
The log–log relationships between the total and soluble concentrations of As (filled circle) and Sb (open circle) in individual soil samples. Solid line (line) represents a linear model for As (*r*^2^ = 0.51, *p* < 0.05). Dashed line (dashed line) represents a linear model for Sb (*r*^2^ = 0.81, *p* < 0.001)

The concentrations of As and Sb in soil water extracts successively increased with the total soil As and Sb (Fig. 3). The water-soluble concentrations of As associated with the site’s processing area and surrounding soils averaged 0.25 ± 0.08 and 0.28 ± 0.12 mg As kg^−1^, respectively, but were significantly higher in spoils (6.30 ± 1.20 mg kg^−1^, *p* < 0.05). In contrast, the highest soluble concentrations of Sb were associated with the site’s ore processing area (up to 24.78 mg kg^−1^). The concentrations of soluble Sb in spoils were significantly lower (1.23 ± 0.23, *p* < 0.05). The lowest soluble Sb concentrations were associated with the surrounding soils (0.06 ± 0.01 mg kg^−1^). The resultant partitioning coefficients varied between 613.75 and 148,719 l kg^−1^ for As and from 30.76 to 1662.39 l kg^−1^ for Sb. The mobility of Sb did not vary spatially (i.e. a similar range of Kd values was calculated for every zone), but the As Kd values associated with the ore processing zone were on average 4 times higher than in spoil heaps and surrounding soils (*p* < 0.05).

Overall, the site’s soil pH (aq.) ranged from acidic to slightly alkaline (3.57 to 7.08) and significantly correlated with the exchangeable acidity of soils (*r*^2^ = 0.95, *p* < 0.05, data not shown). Similarly to As and Sb, there was a significant spatial variation (Table 2). The lowest pH values were associated with the surrounding soils (4.76 ± 0.24). The pH values of soils from the ore sorting area and spoils were higher (5.23 ± 0.48 and 5.61 ± 0.21, respectively); however, only the differences observed for pH of spoil soils were statistically significant (*p* < 0.05). The soil pH negatively correlated with soil OM (*r*^2^ = − 0.55, *p* < 0.05). The highest soil OM values were observed in the ore processing area (up to 10.83%), and the OM values in spoils and surrounding soils were significantly lower (2.51 ± 0.32 and 2.16 ± 0.32%, respectively, *p* ≤ 0.009). The soil pH and OM of samples from the smaller shaft were 4.72 ± 0.48 and 3.03 ± 0.39%, respectively (Table 1). When related to the As and Sb concentrations, soil OM significantly correlated with total Sb in the soils from spoils and ore processing area (*r*^2^ = 0.60, *p* < 0.05), while the soil pH significantly correlated with total As concentrations (*r*^2^ = 0.54, *p* < 0.05). A similar relationship with pH was observed for soluble As (*r*^2^ = 0.49, *p* = 0.002). Soluble As also negatively correlated with soil OM (*r*^2^ = − 0.43, *p* = 0.008). In contrast, no such relationships were identified for soluble Sb.

### Characteristics of water samples

**Fig. 4:**
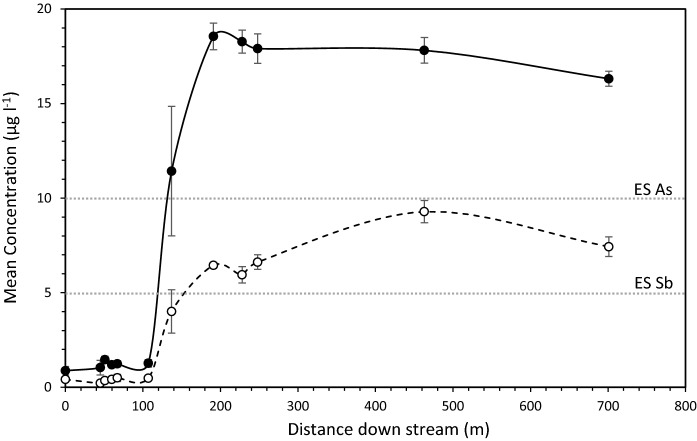
Trends of As (filled circle) and Sb (open circle) concentrations in the Glenshanna Burn with distance from the Louisa mine. Solid line (line) represents the As trend. Dashed line (dashed line) represents the Sb trend. Dotted lines highlight the relevant EU environmental standards for freshwater. The error bars represent a standard error of the calculated mean value (n = 3)

The highest freshwater As concentrations were recorded in the small gully (locations b and c) adjacent to the mine site (average 21.74 ± 0.39 μg l^−1^) (Table 3). The concentrations of As in the samples from the Glenshanna Burn ranged from 0.87 ± 0.22 to 18.55 ± 0.70 μg l^−1^ (Fig. 4). The lowest concentrations were observed upstream of the mine (location a). Following the gully inlet (sampling points d – h), the As concentrations increased to average 1.24 ± 0.09 μg l^−1^ (*p* = 0.008). Approximately 100 m downstream from the mining site (at location i), the As concentrations further increased to 11.43 ± 3.43 μg l^−1^ (*p* < 0.05). The concentrations of Sb followed a similar trend (*r*^2^ = 0.77, *p* < 0.05), albeit no significant increase in Sb concentrations following the gully inlet was observed. The concentrations of Sb significantly increased to 9.28 ± 0.59 μg l^−1^ with As concentrations ~ 100 m downstream of the site (*p* < 0.05) (Fig. 4). The As and Sb concentrations determined at the control site did not exceed 0.47 and 0.36 μg l^−1^, respectively.

**Table 3:** Descriptive statistics of selected chemical parameters of water samples collected from the Glenshanna Burn and gully

Water	Property	Units	Minimum	Maximum	Mean	Standard error	Count
Burn	As	µg l^−1^	0.87	18.55	8.94	2.40	12
	Sb	µg l^−1^	0.21	9.28	3.51	0.99	12
	F	mg l^−1^	0.02	0.05	0.03	0.00	12
	Cl	mg l^−1^	2.41	3.72	3.33	0.09	12
	NO_3_^−^	mg l^−1^	0.19	0.38	0.25	0.02	12
	SO_4_^2−^	mg l^−1^	1.69	2.07	1.88	0.04	12
	DOC	mg l^−1^	4.05	5.69	4.86	0.14	12
	pH		6.58	7.26	7.10	0.06	12
Gully	As	µg l^−1^	21.35	22.13	21.74	0.39	2
	Sb	µg l^−1^	1.69	1.76	1.73	0.04	2
	F	mg l^−1^	0.02	0.02	0.02	0.00	2
	Cl	mg l^−1^	2.02	2.06	2.05	0.02	2
	NO_3_^−^	mg l^−1^	0.24	0.28	0.26	0.02	2
	SO_4_^2−^	mg l^−1^	0.78	0.81	0.79	0.02	2
	DOC	mg l^−1^	5.98	6.16	6.07	0.09	2
	pH		6.52	6.54	6.53	0.01	2

The water pH values varied from slightly acidic to slightly alkaline. The lowest pH value was recorded at the highest sampling location (a) upstream, 6.37 ± 0.07, and the highest pH at the lowest sampling location (n) downstream, 7.26 ± 0.01, gradually increasing between these two locations (Table 3). The pH value of the control samples from the Meggat Water was 7.19 ± 0.02. In contrast, the concentrations of DOC between these two locations (a–n, Fig. 1) successively decreased from 5.85 ± 0.20 mg l^−1^ to 4.05 ± 0.24 mg l^−1^. Both parameters, pH and DOC, were negatively correlated (*r*^2^ = − 0.70, *p* < 0.05). In addition, there was an increase in water SO_4_^2−^ concentrations with distance (*p* < 0.05). No trends were observed for the other analytes (Table 3).

When related to the concentrations of As and Sb in stream water, pH significantly correlated with Sb (*r*^2^ = 0.67, *p* < 0.05), but not with As. The concentrations of DOC were significantly negatively related to both As and Sb (*r*^2^ = − 0.81 and *r*^2^ = − 0.90, respectively, *p* < 0.05). In contrast, SO_4_^2−^ significantly increased with As and Sb in water samples (*r*^2^ = 0.71 and *r*^2^ = 0.68, respectively, *p* < 0.05).

### Environmental risk and pollution of the site

**Fig. 5:**
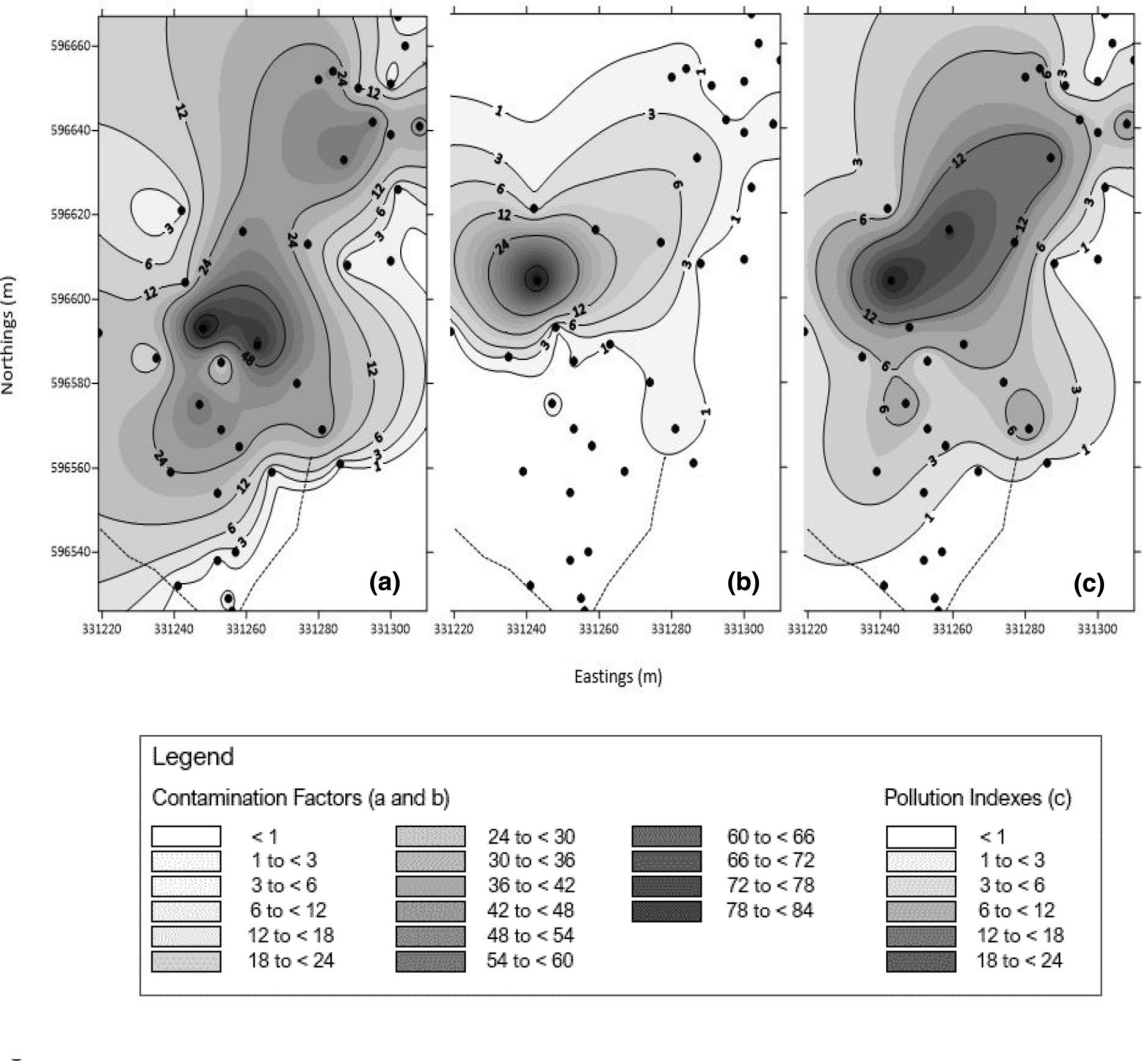
The spatial distribution of site’s contamination based on CF calculated for As (**a**) and Sb (**b**). The overall pollution of the site summarised by IPI is presented in section (**c**). The greyscale signifies the increase in As (CF from 1 to 82.84) and Sb contamination (CF from 1 to 60.17) and overall pollution (IPI from 1 to 24.75)

Exceedances of EU environmental standards by both elements ~ 100 metres downstream of the site are indicated in Fig. 4. In both cases, the highest concentrations of As and Sb detected in the Glenshanna Burn were almost double the levels permitted in the European freshwaters (10 and 5 µg l^−1^, respectively). The samples taken from the gully only exceeded the freshwater standards for As (Table 3). Further, the generic risk assessment of total soil concentrations revealed that all As concentrations greatly exceeded the soil threshold of 50 mg kg^−1^. In contrast, the exceedance of Sb threshold (10 mg kg^−1^) was limited to soils associated with spoils and ore processing area (Table 2). The contamination factors calculated for As ranged from 0.58 to 82.84 (unitless), with spoils and ore processing area being highly contaminated (maximum CF = 82.84 and 32.95, respectively) (Fig. 5a). In contrast, the highest Sb contamination (maximum CF = 60.17) was associated with the ore processing area (Fig. 5b). The Sb contamination associated with the spoils was moderate (average CF 1.39 ± 0.27). The soils surrounding the site were not contaminated with Sb (average CF = 0.15 ± 0.02), but indicated a moderate As contamination (average CF = 1.93 ± 0.37). The IPI analysis revealed extreme pollution of soils sampled from the spoils and ore processing area (Fig. 5c). However, the IPI values derived for the ore processing area were significantly higher (*p* < 0.05). The soils surrounding the site were not polluted (average IPI = 0.51 ± 0.08).

## Discussion

The concentrations of As and Sb in soils around the Louisa mine are geochemically elevated (up to 350 and 40 mg kg^−1^, respectively) due to underlying geology (Gallagher et al. 1983). These concentrations are comparable to As and Sb concentrations in the European soils (< 2 to ~ 300 and < 0.5 to ~ 30 mg kg ^−1^, respectively) detailed by Tóth et al. (2016). The mining activities, however, have been reported to increase such values up to three orders of magnitude (Hiller et al. 2012; Warnken et al. 2017). The concentration ranges of As and Sb found in this study (Table 1) corresponded to the range of values (50–17,400 and 10–1200 mg kg^−1^ for As and Sb, respectively) previously reported for the site by Gál et al. (2007). In comparison with Macgregor et al. (2015) and Flynn et al. (2003), the concentrations of Sb were generally lower (with the exception of 1504.17 mg kg^−1^ at point 15), while the concentrations of As were considerably higher. This disparity with some of the earlier studies of the site, however, was not surprising, considering the large spatial variation of As and Sb concentrations across the site (Table 1 and Table 2). The anthropogenic redistribution of As and Sb within the site altered the natural As and Sb ratio observed in the surrounding soils (~ 100:1, respectively) (Protano and Nannoni 2018). Consequently, the concentrations of As across the site differed by more than 10,000 mg kg^−1^ (Gál et al. 2007). In agreement with the studies of similar antimony mines, the highest concentrations of As were in spoil heaps (the spent material abundant in arsenopyrite, with the As to Sb ratio of ~ 300:1) (Fu et al. 2016; Hiller et al. 2012; Warnken et al. 2017). In contrast, the high Sb concentrations associated with the processing area were sourced from stibnite containing ores handled within this zone (Protano and Nannoni 2018). The concentrations of both, As and Sb, subsequently decreased with distance from these zones. For instance, for the soils collected between the spoil heap and Glenshanna Burn (points 33–37, Fig. 2) a sharp decrease in both As and Sb concentrations was recorded. This confirmed the findings of Macgregor et al. (2015) and suggested that the site’s contamination does not readily diffuse into the surrounding soils.

The investigation of As and Sb mobility in soils did not indicate any significant differences in Sb partitioning coefficients across the site (average 233.44 ± 49.06 l kg^−1^). As previously described by Protano and Nannoni (2018) and Tan et al. (2018), the concentrations of soluble Sb steadily increased with the total soil Sb (Fig. 3), but significantly surpassed the value of < 1% usually reported (Álvarez-Ayuso et al. 2012; Müller et al. 2007). Nevertheless, the observed trends for soluble Sb tend to be reported for exchangeable fraction (Nannoni et al. 2011) and have been linked to Sb association with Sb(Fe) or Fe(Sb) oxides/hydroxides around silicate grains and with organic matter (Gál et al. 2007; Hiller et al. 2012). The soluble and exchangeable As was reported to form similar associations with Fe oxides (Abad-Valle et al. 2018), but the derived average As partitioning coefficient was two orders of magnitude higher (~ 20,727 l kg^−1^) than for Sb. The partitioning coefficients reported for As vary (De Brouwere et al. 2004), but the soluble As fractions of < 1% confirmed the findings of Gál et al. (2007) and were comparable to the results of other studies (Nannoni et al. 2011; Tan et al. 2018). Although As appeared to be proportionally more retained by soils, neither of the elements could be termed as easily released by soils with an apparent threat to the surrounding environment.

Despite the determined partitioning coefficients indicating limited As and Sb release from soils, the concentrations of As potentially released by the soils were of environmental concern. For example, the soluble As concentrations observed in the ore processing area were comparable to those of the surrounding soils and findings of Álvarez-Ayuso et al. (2012), but for spoil heaps were 25 times higher (6.29 ± 1.20 mg kg^−1^). Such high concentrations represent a considerable threat to pasture (Abad-Valle et al. 2018), as the limit recommended for soluble As in agricultural soils is 0.04 mg kg^−1^ (Álvarez-Ayuso et al. 2012). Further, the soluble As concentrations in water extracts (1573.73 ± 299.19 µg l^−1^) also exceeded the EU limit values for non-hazardous waste leachate (200 µg l^−1^) (Ettler and Mihaljevic 2010). The same applied to the Sb concentrations in water extracts from the spoil heaps and ore processing area (up to 1119.12 and 6195.97 µg l^−1^, respectively). While these values were comparable to Hiller et al. (2012), they exceeded the EU limits of 70 µg l^−1^ for non-hazardous waste (Ettler et al. 2010).

The threat associated with the uptake and/or leaching of As and Sb from soils is dictated by the soil physico-chemical properties (De Brouwere et al. 2004). The soils around the site have been described as acidic, aerobic and containing Fe oxides and oxyhydroxides (Gál et al. 2007), which strongly sorb Sb (Fillela et al. 2009; Nannoni et al. 2011). But mobility of Sb is known to increase with increasing soil pH (Wilson et al., 2010). Any such increase in Sb mobility in spoils and ore processing area, however, was in this study obscured by Sb association with increasing soil OM (Flynn et al. 2003; Gál et al. 2007; Macgregor et al. 2015). Consequently, the main factor controlling the release of Sb from soils was the total soil Sb concentration (Fig. 3).

Compared to Sb, the sorption of As to soil OM tends to be weaker, while the effect of pH is more pronounced (De Brouwere et al. 2003; Wilson et al. 2010). In this study, the soluble As decreased with soil OM, but increased with the increasing soil pH. The finding agrees with the observations of arsenopyrite weathering to scorodite (FeAsO_4_·2H_2_O) and its further dissolution with increasing pH (Abad-Valle et al. 2018). This widely recognised phenomenon releases As(V) into the deeper layers of spoil heaps, where reducing conditions promote formation of As(III) in place of As(V) (Smedley and Kinniburgh 2002). The change in As speciation results in lower sorption and build-up of As concentrations at the base of spoil heaps (Fu et al. 2016). The increase in As concentrations with the depth of spoil heaps at the Louisa mine was confirmed by Macgregor et al. (2015). The build-up in As concentrations can subsequently lead to unconstrained leaching into the surrounding environment (Abad-Valle et al. 2018).

The water samples collected from the gully provided the highest concentrations of dissolved As (up to 22.13 ± 0.98 μg l^−1^), double the EU ES (Ondrejková et al. 2013), but significantly lower than 1770 μg l^−1^ reported by Macgregor et al. (2015). The concentrations of Sb were also much lower than 783 μg l^−1^ reported by the same author, but significantly above the background concentrations of < 1 μg l^−1^ associated with the unimpacted freshwater (Wang et al. 2011). The observed disparity has been explained by Fillela et al. (2009), who observed a high variation in Sb concentrations over time caused by rain events and associated fluctuations in water flow. The relative ratio of As vs. Sb in the gully run-off of 12:1 directly related these values to the concentrations of soluble As and Sb present at the same ratio in the adjacent spoil heap (Lower spoil, Table 2).

The inflow of gully water into the Glenshanna burn significantly increased the stream water concentrations of As and Sb, as well as their natural ratio (from 2:1 to 5:1, respectively). Further downstream (~ 65 m, location g), the As/Sb ratio returned to 2:1 indicating that the impact of the gully drainage on the Glenshanna Burn is minimised through dilution (Macgregor et al. 2015; Ritchie et al. 2013). In contrast, the concentrations of As and Sb approximately 100 m downstream of the site yielded the same As and Sb ratios as the upstream samples, despite being twice the EU environmental standard for the freshwater (Fig. 4). This is consistent with the findings of Ritchie et al. (2013), who observed an increase in As and Sb concentrations in stream water from geochemically mineralised areas. The strata below the Louisa mine has been intensely folded and faulted, with exposed geochemically mineralised zones (Gallagher et al. 1983; Macgregor et al. 2015). The interaction of stream water with these zones and their natural weathering can solubilise As and Sb (Smedley and Kinniburgh 2002). For example, Borčinová Radková et al. (2020) described that the weathering of stibnite, the main mineral associated with the Louisa mine, in near-surface (oxidising) conditions releases As and Sb. Antimony is released in Sb(III) form, but is quickly oxidised to Sb (V). Arsenic undergoes the same oxidation process, but remains in its As(III) form for longer (Borčinová Radková et al. 2020; Fu et al. 2016). In freshwater, both released elements predominantly exist in their As(V) and Sb(V) states. While this is a generally supported assumption, Fu et al. (2016) demonstrated that As and Sb speciation is dictated by environmental characteristics, particularly pH. Although this study aligns with the findings of similar studies, such as Barats et al. (2017) or Fillela et al. (2009), the geochemical processes causing the As and Sb enrichment of the Glenshanna Burn should be further investigated.

## Conclusions

Overall, the site of the former Louisa mine has been found grossly contaminated by Sb and As from the mining activities (Fig. 5a, b). Both, As and Sb exceed the relevant soil thresholds, indicating that this could pose a human and environmental health hazard (Guo et al. 2017). The assessment of pollution highlighted two main pollution sources—the spoil heaps and the ore processing area (Fig. 5c). The soil pollution of the ore processing area was associated with very high soluble Sb concentrations, but its transport is limited by the nature of the underlying geology. The vertical and steeply dipping strata combined with quartz veins, mudstones and shales present an impenetrable barrier preventing the lateral migration of fluids down the slope towards the Glenshanna Burn. Further, the physico-chemical properties of spoil heaps limit the Sb mobility, while promoting the mobility of As. The soluble concentrations of As associated with the lower spoil heap have an impact on the Glenshanna Burn, due to a surface run-off of contaminated leachates through the gully. The gully represents a direct pathway between the lower spoil heap and the Glenshanna Burn. The current impact of the leachate on the Glenshanna Burn is mitigated by dilution. As such, the surface run-off does not cause an exceedance of freshwater environmental standards. However, as the physico-chemical conditions of spoil heaps evolve towards reducing environment, it might exacerbate the associated impacts. In contrast, the exposed bedrock, which the Glenshanna Burn flows through, is a geochemical source of As and Sb, causing the exceedance of the EU environmental standards. The extent of this type of pollution in the Glenshanna Burn and its potential impact on the residents of Jamestown, however, requires further study.

This study focussed on a defined catchment in Southern Scotland, but the findings are widely transferrable. Numerous antimony mining sites around the world, both derelict and operational, share similarities with the Louisa mine and directly impact water quality. The risks associated with geogenic sources of contamination should not be overlooked. The methodology adopted by this study was able to differentiate anthropogenically polluted soil zones and geochemically enriched surface water. This approach revealed that to competently mitigate receptor exposure there is a need to comprehensively understand the source and form of elemental contamination.
